# Expression of plasma levels of matrix metalloproteinases and their inhibitors in patients with abdominal aortic aneurysm

**DOI:** 10.1590/1677-5449.202501352

**Published:** 2026-01-09

**Authors:** Vanessa Souza Silva Medrado, Domingos Sávio de Oliveira e Silva, Leonardo Alves Santos, Luana Macedo da Silva Nascimento, Rodrigo Mendes, Pedro Pereira Tenório

**Affiliations:** 1 Universidade Federal do Vale do São Francisco UNIVASF, Paulo Afonso, BA, Brasil.; 2 Universidade Federal de São Paulo UNIFESP, Escola Paulista de Medicina, São Paulo, SP, Brasil.; 3 Universidade Federal do Vale do São Francisco UNIVASF, Paulo Afonso, BA, Brasil.

Ao Editor,

Li a carta “Expressão dos níveis plasmáticos de metaloproteinases e seus inibidores em pacientes com aneurisma da aorta abdominal”. Em primeiro lugar, agradeço as observações, e tenho alguns comentários a fazer.

Diante da obrigação de vigilância dos pacientes submetidos ao tratamento endovascular de aneurisma de aorta abdominal (AAA), há necessidade de se desenvolverem métodos não invasivos, a fim de identificar os *endoleaks* e indicar intervenção precoce. Atualmente, os exames de vigilância ainda são dependentes do diagnóstico imagiológico do médico observador, bem como da plataforma de análise e mensuração do exame.

As metaloproteinases de matriz (MMPs) constituem uma família de enzimas com atividade proteolítica geneticamente relacionadas, cuja atividade é dependente de zinco. O tecido aneurismático caracteriza-se pela elevação da atividade elastolítica e colagenolítica, resultante do aumento da produção de enzimas proteolíticas em comparação ao tecido aórtico normal. Na presente casuística, não houve diferença entre as atividades de MMP-2 e MMP-9 após 6 meses de pós-operatório entre pacientes com e sem *endoleak* nesse período. Entretanto, houve aumento das MMP-2 e MMP-9 antes e após o tratamento endovascular^1^.

As razões para essa discrepância podem, em parte, estar relacionadas a diferenças nas populações estudadas, ao manuseio distinto de amostras de sangue, à avaliação dos métodos ou à permanência do tecido aneurismático. Realmente, o viés relacionado a outras doenças não vasculares, ou até mesmo à própria aterosclerose, podem ser fatores de imprecisão dos resultados dos biomarcadores. Identificar uma maneira de mitigar o efeito confundidor traria uma compreensão mais fornida da fisiopatologia dos aneurismas, assim como melhor sensibilidade e especificidade no *screening* de *endoleaks*. Portanto, estudos multicêntricos que incorporem diferentes marcas de endopróteses, e, se possível, a comparação com o tratamento cirúrgico aberto, poderiam fornecer mais informações sobre a fisiopatologia e o manejo completo com exclusão do saco aneurismático, além de permitir um seguimento mais prolongado da dosagem desses marcadores.

Em outra publicação de Leite et al.^2^, os miR-21 e miR-181b foram identificados no sangue total de pacientes sem aneurismas, com AAA e com *endoleak*. Observou-se aumento significativo de suas expressões antes do tratamento endovascular, quando comparados ao grupo controle. Após 6 meses do procedimento, verificou-se redução significativa dessas expressões em relação ao grupo estudo, sugerindo que a exclusão do saco aneurismático altera a expressão dos miRNAs. Entretanto, entre os pacientes que apresentaram *endoleak*, não houve diferença estatística. Adicionalmente, as expressões de miR-21 e miR-181b não demonstraram correlação com a anatomia, o diâmetro do aneurisma ou os diferentes tipos de endopróteses utilizados no tratamento endovascular dos AAAs.

Diante da cascata de reações relacionadas ao miR-21, balões expansíveis ou endopróteses com eluição de drogas contendo pré-miR-21 poderiam representar uma ferramenta promissora para estimular a proliferação celular na parede da aorta em pacientes com AAA. Em relação ao miR-181b, a utilização de uma droga anti-miR-181b reduz a expressão de SIRT1 (sirtuína 1) e HO-1 (heme oxigenasse-1), promovendo maior elasticidade e menor rigidez da parede vascular, o que também poderia configurar uma estratégia terapêutica promissora para pacientes com fatores de risco para aterosclerose e aneurismas^2^.

Portanto, embora o potencial clínico dos biomarcadores seja promissor, ainda existem desafios a serem superados antes de sua implementação prática. Entre eles, destacam-se: a necessidade de padronização dos métodos, com o desenvolvimento de protocolos uniformes para coleta, processamento e análise de miRNA, MMP e TIMP; a validação clínica em estudos multicêntricos de larga escala; além da integração com a prática clínica e da garantia de acessibilidade a todos os pacientes, com ferramentas viáveis para aplicação rotineira.

Dear Editor,

We read with great interest the article by Leite et al.,^1^ entitled “Expression of plasma levels of MMP-2, MMP-9, TIMP-1, and TIMP-2 in patients with abdominal aortic aneurysms”, published in *Jornal Vascular Brasileiro*. The study addresses a highly relevant topic in vascular surgery, particularly regarding the role of circulating biomarkers in the follow-up after endovascular aneurysm repair (EVAR). The authors deserve recognition for the prospective design, the 6-month follow-up, and the inclusion of a control group, which contributes to the study’s methodological robustness. The demonstration of increased levels of matrix metalloproteinases (MMPs) and tissue inhibitors of metalloproteinases (TIMPs) after EVAR, regardless of the presence of an endoleak, provides an important perspective on the biological response to endovascular aneurysm exclusion. As previously pointed out by McMillan and Pearce,^2^ elevated levels of MMPs are associated with aortic wall degradation, and the present study extends this knowledge to post-EVAR context.

The authors also emphasize, in line with Hellenthal et al.,^3^ that plasma biomarkers still have limitations in diagnostic accuracy for detecting endoleaks, with computed tomography angiography remaining the primary imaging modality for follow-up. However, the observation that MMP-2 and TIMP-1 increased significantly even in the absence of endoleaks suggests that these markers may reflect an inflammatory or remodeling process of the aortic wall, rather than solely the persistence of aneurysmal sac pressurization. An interesting aspect of the study by Leite et al.^1^ was the use of 8 different types of endografts, which may induce variability in the systemic inflammatory response but also reflects real world clinical practice and reinforces the external applicability of the findings. The analysis of potential differences in inflammatory response among the models, although not statistically significant, could be further explored in future studies.

Despite the relevant contributions, some points warrant reflection and further refinement. A longer follow-up period (beyond 6 months) could clarify whether the elevations of these biomarkers persist or normalize, and whether they are associated with late endoleaks or aneurysm sac enlargement. Controlling confounding factors, such as atherosclerosis and chronic inflammation, would allow for greater specificity in the interpretation of the results. It is worth noting that conditions such as atherosclerosis, autoimmune diseases, neoplasms, and the use of anti-inflammatory medications may also influence MMP and TIMP levels. The lack of control for these variables, as acknowledged by the authors themselves, limits the accuracy of the association between the biomarkers and EVAR per se. Although the number of patients is adequate, the low number of observed endoleaks (n = 10) limits the statistical power for more detailed analyses. Therefore, it would also be of interest to investigate whether the different types of endografts used influence the inflammatory response and biomarker levels, considering variations in radial force and sealing mechanisms.

In summary, the study by Leite et al. represents an important contribution to the literature on molecular follow-up after EVAR. The findings reinforce the need for new multicenter studies with larger samples, longer follow-up, and the incorporation of emerging biomarkers, such as miRNAs and inflammatory cytokines (Tenorio et al.),^4^ which may improve postoperative surveillance.

Dear Editor,

I have read the letter entitled “Expression of plasma levels of matrix metalloproteinases and their inhibitors in patients with abdominal aortic aneurysm.” First, I would like to thank you for your observations, and I would like to make a few comments.

Given the obligation to monitor patients undergoing endovascular treatment of abdominal aortic aneurysm (AAA), there is a need to develop non-invasive methods to identify endoleaks and indicate early intervention. Currently, surveillance examinations still depend on the imaging diagnosis made by the physician, as well as on the analysis and measurement platform used.

Matrix metalloproteinases (MMPs) constitute a family of genetically related enzymes with zinc dependent proteolytic activity. Aneurysmal tissue is characterized by increased elastolytic and collagenolytic activity, resulting from the enhanced production of proteolytic enzymes compared with normal aortic tissue. In the present series, no difference was observed in MMP-2 and MMP-9 activity at 6 months postoperatively between patients with and without endoleak during this period. However, there was an increase in MMP-2 and MMP-9 levels before and after endovascular treatment.^1^

The reasons for this discrepancy may be related to differences in the populations studied, to variations in blood sample handling, to methodological assessment, or to the persistence of aneurysmal tissue. Indeed, bias related to other non-vascular diseases, or even to atherosclerosis itself, may contribute to imprecision in biomarker results. Identifying a way to mitigate the confounding effect would provide a more comprehensive understanding of aneurysm pathophysiology, as well as improved sensitivity and specificity in endoleak screening. Therefore, multicenter studies that incorporate different brands of endografts and, if possible, comparisons with open surgical repair could provide further insights into the pathophysiology and comprehensive management with exclusion of the aneurysmal sac, in addition to allowing longer term follow-up of biomarker levels.

In another publication by Leite et al.,^2^ miR-21 and miR-181b were identified in whole blood from patients without aneurysms, with AAA, and with endoleak. A significant increase in their expression was observed before endovascular treatment when compared with the control group. After 6 months of the procedure, a significant reduction in these expressions was observed in the study group, suggesting that exclusion of the aneurysmal sac alters miRNA expression. However, among patients who presented with endoleak, no statistical difference was found. Additionally, the expressions of miR-21 and miR-181b showed no correlation with anatomy, aneurysm diameter, or the different types of endografts used in the endovascular treatment of AAA.

Given the cascade of reactions related to miR-21, expandable balloons or drug-eluting endografts containing pre-miR-21 could represent a promising tool to stimulate cell proliferation in the aortic wall of patients with AAA. Regarding miR-181b, the use of an anti-miR-181b drug reduces the expression of SIRT1 (sirtuin 1) and HO-1 (heme oxygenase-1), promoting greater elasticity and reduced stiffness of the vascular wall, which could also represent a promising therapeutic strategy for patients with risk factors for atherosclerosis and aneurysms.^2^

Therefore, although the clinical potential of biomarkers is promising, there are still challenges to be overcome before their practical implementation. Among these, the following stand out: the need for standardization of methods, with the development of uniform protocols for the collection, processing, and analysis of miRNA, MMP, and TIMP; clinical validation in large-scale multicenter studies; as well as integration into clinical practice and assurance of accessibility for all patients, with feasible tools for routine application.

## Data Availability

Todos os dados gerados ou analisados estão incluídos neste artigo e/ou no material suplementar.
