# Supplementary material for: Expression of plasma levels of matrix metalloproteinases and their inhibitors in patients with abdominal aortic aneurysm
Source: J Vasc Bras. 2026 Jan 9;24:e20250135. [Article in Portuguese] doi: 10.1590/1677-5449.202501352 (PMC12974594; doi:10.1590/1677-5449.202501352)
Supplement: Translation

# Translation: Expressão dos níveis plasmáticos de metaloproteinases e seus inibidores em pacientes com aneurisma da aorta abdominal

**DOI:** 10.1590/1677-5449.202501351

**Authors:** Vanessa Souza Silva Medrado, Domingos Sávio de Oliveira e Silva, Leonardo Alves Santos, Luana Macedo da Silva Nascimento, Rodrigo Mendes, Pedro Pereira Tenório

**Affiliations:** 1 Universidade Federal do Vale do São Francisco UNIVASF, Paulo Afonso, BA, Brasil.; 2 Universidade Federal de São Paulo UNIFESP, Escola Paulista de Medicina, São Paulo, SP, Brasil.; 3 Universidade Federal do Vale do São Francisco UNIVASF, Paulo Afonso, BA, Brasil.

Ao Editor,

Lemos com grande interesse o artigo de Leite et al.^1^, intitulado “Expressão dos níveis plasmáticos de MMP-2, MMP-9, TIMP-1 e TIMP-2 em pacientes com aneurisma da aorta abdominal”, publicado no Jornal Vascular Brasileiro. O estudo aborda uma temática de elevada relevância na cirurgia vascular, especialmente no que diz respeito ao papel de biomarcadores circulantes no seguimento pós-reparo endovascular de aneurismas (EVAR). Os autores merecem reconhecimento pelo desenho prospectivo, pelo acompanhamento de 6 meses e pela inclusão de um grupo controle, que contribui para a robustez metodológica do trabalho. A demonstração de níveis aumentados de metaloproteinases de matriz (MMPs) e inibidores teciduais de metaloproteinases (TIMPs) após EVAR, independentemente da presença de *endoleak*, oferece uma importante perspectiva sobre a resposta biológica à exclusão endovascular do aneurisma. Conforme já apontado por McMillan e Pearce^2^, níveis elevados de MMPs estão associados à degradação da parede aórtica, e o presente estudo amplia esse conhecimento para o contexto pós-EVAR.

Os autores também reforçam, em consonância com Hellenthal et al.^3^, que biomarcadores plasmáticos ainda possuem limitações na acurácia diagnóstica para detecção de *endoleaks*, sendo a angiotomografia computadorizada o principal exame de imagem de acompanhamento. No entanto, a observação de que MMP-2 e TIMP-1 aumentaram significativamente, mesmo na ausência de *endoleaks*, sugere que esses marcadores podem refletir um processo inflamatório ou de remodelamento da parede aórtica, e não apenas a persistência de pressurização do saco aneurismático. Um ponto interessante do estudo de Leite et al.^1^ foi a utilização de oito tipos distintos de endopróteses, o que pode induzir variabilidade na resposta inflamatória sistêmica, mas também reflete a prática clínica real e reforça a aplicabilidade externa dos achados. A análise de eventuais diferenças de resposta inflamatória entre os modelos, embora não tenha alcançado significância estatística, poderia ser ampliada em estudos futuros.

Apesar das contribuições relevantes, alguns pontos merecem reflexão e aprimoramento. Um acompanhamento mais prolongado (além de 6 meses) poderia esclarecer se as elevações desses biomarcadores persistem ou se normalizam, e se estão associadas à *endoleaks* tardios ou ao aumento do saco. O controle de fatores de confusão, como aterosclerose e inflamação crônica, sobretudo, permitiria melhor especificidade na interpretação dos resultados. Cabe destacar que condições como aterosclerose, doenças autoimunes, neoplasias e uso de medicamentos anti-inflamatórios também podem influenciar os níveis de MMPs e TIMPs. A ausência de controle para essas variáveis, apontada pelos próprios autores, limita a precisão da associação entre os biomarcadores e o EVAR em si. Embora o número de pacientes seja adequado, o baixo número de *endoleaks* observados (n = 10) limita o poder estatístico para análises mais detalhadas. Assim, seria interessante investigar também se diferentes tipos de endopróteses utilizados influenciam a resposta inflamatória e os níveis de biomarcadores, considerando variações na força radial e nos mecanismos de vedação.

Em suma, o trabalho de Leite et al. constitui uma importante contribuição à literatura sobre o seguimento molecular após EVAR. Os achados reforçam a necessidade de novos estudos multicêntricos, com amostras maiores, acompanhamento prolongado e incorporação de biomarcadores emergentes, como miRNAs e citocinas inflamatórias (Tenorio et al.)^4^, que podem melhorar a vigilância pós-operatória.

## Data Availability

All data generated or analysed during this study are included in this published article and/or its supplementary material.
